# Differential Response of Fish Assemblages to Coral Reef-Based Seaweed Farming

**DOI:** 10.1371/journal.pone.0118838

**Published:** 2015-03-30

**Authors:** E. James Hehre, J. J. Meeuwig

**Affiliations:** 1 Sea Around Us Project /Fisheries Center, University of British Columbia, Vancouver, BC, Canada; 2 School of Animal Biology and Centre for Marine Futures (Oceans Institute), University of Western Australia, Crawley, WA, Australia; ufrj, BRAZIL

## Abstract

As the global demand for seaweed-derived products drives the expansion of seaweed farming onto shallow coral ecosystems, the effects of farms on fish assemblages remain largely unexplored. Shallow coral reefs provide food and shelter for highly diverse fish assemblages but are increasingly modified by anthropogenic activities. We hypothesized that the introduction of seaweed farms into degraded shallow coral reefs had potential to generate ecological benefits for fish by adding structural complexity and a possible food source. We conducted 210 transects at 14 locations, with sampling stratified across seaweed farms and sites adjacent to and distant from farms. At a seascape scale, locations were classified by their level of exposure to human disturbance. We compared sites where (1) marine protected areas (MPAs) were established, (2) neither MPAs nor blast fishing was present (hence “unprotected”), and (3) blast fishing occurred. We observed 80,186 fish representing 148 species from 38 families. The negative effects of seaweed farms on fish assemblages appeared stronger in the absence of blast fishing and were strongest when MPAs were present, likely reflecting the positive influence of the MPAs on fish within them. Species differentiating fish assemblages with respect to seaweed farming and disturbance were typically small but also included two key target species. The propensity for seaweed farms to increase fish diversity, abundance, and biomass is limited and may reduce MPA benefits. We suggest that careful consideration be given to the placement of seaweed farms relative to MPAs.

## Introduction

Seascapes are being transformed on a global scale [1] with human activities creating mosaics of modified habitat. This is particularly true for spatially extensive, extractive activities like fishing which result in dwindling residual areas of marine wilderness [2,3]. There is a general recognition that changes in activities can lead to ecosystem impacts, but the nature of these impacts on the regional ecology remain difficult to predict [1,2]. Additionally, while many studies have focused on the last remnants of wild nature in order to preserve them [3–6], “novel” ecosystems are increasingly recognized as a consequence of changing species assemblages resulting from climate and land use change [7]. Indeed, recent research has documented the connection between the intensity of human activity and subsequent changes in ecosystems, including declines in diversity [8–12], occurrence of invasive species [13–15], or habitat homogenization [16–19]. This suggests that over time, novel ecosystems will become increasingly extensive over large areas of the world [7].

The transformation of seascapes has significant implications for fish communities since fish assemblage structure is strongly correlated to habitat [20–23]. The introduction of new human activities that alter fishing mortality or habitat [21,42] can lead to changes in species diversity [8–12], abundance [24,25], and biomass [26], as well as changes in community composition [27], size structure [28], and distribution [29] of fish assemblages. The degree to which these anthropogenic activities affect fish will be related to the nature of both the habitat and the activities. For instance, in less impacted shallow coral reef ecosystems, where the percentage of living coral is relatively high compared to dead coral or rubble, coral cover decreases with increasing human presence through the combined effects of trampling, shading, siltation, and mechanical damage [8]. Conversely, in highly disturbed areas where the majority of coral is already dead, the impacts/influence of any additional human disturbance may not be detectable. In fact, it is conceivable that human presence may serve to benefit underlying benthos in some cases by reducing some of the most destructive activities, like blast and cyanide fishing, and replacing them with less destructive ones[8].

Seaweed farming provides a useful case study for the creation of novel ecosystems through the addition of new, potentially less destructive human activities [7] within already degraded environments because of both its scale and ubiquity. Commercial harvesting occurs in approximately 35 countries around the world in waters ranging from cold temperate to tropical, providing a variety of products that, in 2008, had a total annual value of US$7.35 billion [30]. In the Indo-Pacific region, seaweed farming consists mostly of small subsistence farms (~1 ha^2^), and their proliferation is in large part governed by both accessibility to useable habitats and proximity to markets [8]. As demand for seaweed derived products increases, farms once primarily located on shallow seagrass beds [10,12,31–33] are now expanding into new locations that consist almost exclusively of shallow coral reefs [31]. In addition, multinational corporations are converting large areas of patchy small community farms into extensive industrial-scale ventures [17]. The potential ecosystem impacts of this expansion include loss of coral cover through increased siltation, trampling, shading, and impairment of recruitment ability [8]. The subsequent impacts on fish communities, as well as the potential for direct disturbance, may cause further declines in fish diversity and abundance, with important implications for food security.

Little information currently exists on the ecological impacts of seaweed production on shallow coral reef ecosystems or the fish assemblages they support. Indeed, previous research has solely focused on single facets of farm impacts such as shading, siltation, and mechanical damage, and has been conducted primarily within seagrass beds, typically at the level of individual farms [8,10,12]. However, shallow coral reef ecosystems are important biologically and socioeconomically; they are hotspots of diversity and productivity, maintain protective barriers for coastlines, and provide a source of livelihood and sustenance to over a million small-scale fishers [13].

Here, we investigated the impact of seaweed farming on fish assemblages in a rare shallow double barrier reef ecosystem, the Danajon Bank of the Philippines. The Philippines is the third largest producer of farmed seaweeds internationally [34], and farming on the Danajon Bank is a growing industry that is expanding rapidly across the entire system [8,17]. We hypothesized that in degraded coral ecosystems, seaweed farming would have a positive effect on the species richness, abundance, and overall biomass of fish assemblages as it adds structural complexity and food to the habitat [8]. As seaweed farms may also be located near marine protected areas (MPAs) and/or be exposed to blast fishing, we were additionally interested in the effects that the level of nearby protection/disturbance may have on the relationship between seaweed farms and fish assemblages. Specifically, we tested whether locations with well-enforced MPAs (where disturbance was relatively low), would have higher diversity, abundance, and biomass due to both benefits from the seaweed farms and spillover effects from the MPAs. In locations subject to blast fishing (and thereby more highly disturbed), seaweed farms may function as *de facto* MPAs, augmenting fish diversity, abundance, and biomass.

## Methods

### 2.1 Study area

The Philippines is located in the heart of the Coral Triangle, an area that encompasses the Indonesia-Philippines and the Southwestern Pacific biogeographic regions, and is widely considered to be the global epicenter of marine biodiversity [35]. Situated off the northern shore of Bohol Island, Danajon Bank is the only double barrier reef in the Philippines and one of only three such reefs in the Indo-Pacific region [36]. The reef stretches over a total area of 2,353 km^2^ comprising 40 islands and over 700 km of aggregate coastline, and represents 1% of the 27,000 km^2^ of estimated total coral reef cover in the Philippines.

Most seaweed farming on the Danajon Bank is of *Eucheuma spinosum* and *Kappaphycus alvarezii* and is practiced on an artisanal scale, although Taiwanese and South Korean interests operate a number of large-scale industrial farms. While several different farming techniques are employed on the Danajon Bank, *E*. *spinosum* is produced primarily through a broadcast method whereby seedlings are cast out onto the shallow coral and harvested at a later date. In contrast, *K*. *alvarezii* is primarily farmed by attaching seedlings to nylon monolines anchored with a series of mangrove stakes on coral substrate generally laid parallel to the reef situated in shallow water between the reef drop off and the shoreline. These two methods were the only ones encountered and co-occurred at each of the study sites. As monoline farms are clearly delineated by stakes, they were the focus of our study. The depth of these farms varied between a few centimeters to 1 m at mean low tide to ensure propagules were not exposed during low water.

Blast fishing is still common in some communities on the Danajon Bank (Hehre pers. obs.), although its exact history within the study area is difficult to determine. Local oral histories date the genesis of blast fishing to the late 1950s or early 1960s. This aligns with the consensus of experts in the Philippines Bureau of Fisheries and Aquatic Resources (BFAR) as well as several regional NGOs that blast fishing in this area has been chronic over a long period of time. The presence of cratering indicates that blast fishing has contributed to the extensive rubble fields observed. Our own estimates, based on the advanced weathering of the rubble, places some of the damage to be several decades old and as witnessed, the practice continues to this day. Regardless of the time frame, extensive blast fishing is most likely the cause of the general homogenization of habitat within some locations, and may potentially impede coral recovery for decades if not centuries (Fox et al 2003; Fox and Caldwell 2006).

### 2.2 Sampling

**Fig 1:**
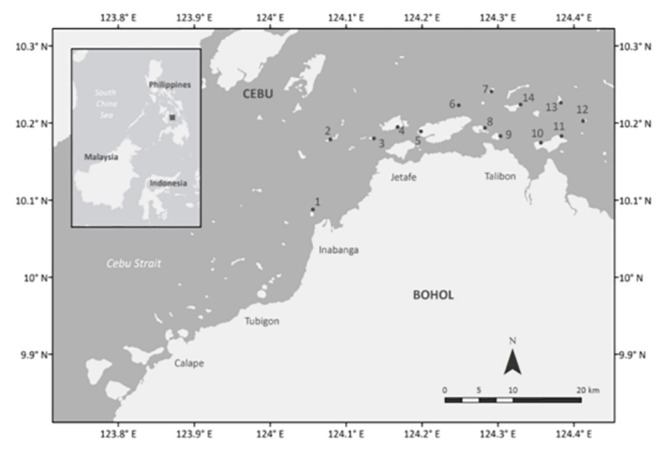
Map of the study area, showing the location of sampling sites. (1) Pandao, (2) Pandanon, (3) Jandayan Sur. (4) Jandayan Norte, (5) Handumon, (6) Tahong Tahong, (7) Guindacpan, (8) Tambo, (9)Banbanon, (10) Busili-an, (11)Pinamgo, (12) Cataban, (13) Saag, (14) Bansaan

Fish assemblages were first sampled at Handumon between mid-June and the end of July in 2010. Following this pilot work, the remaining 13 locations (~ 2.3 km^2^ each) were sampled from mid-June to mid-September, 2011 (Fig 1; Table 1). All sampling was done using standard underwater visual fish census (UVC) [37]. At the 14 locations, sampling sites (<2500 m^2^ each) were established (1) within the seaweed farm (SF), (2) adjacent (ADJ) to the farm (but no further than 5 m from the farm edge) and (3) at a distance (FAR) from the farm (at least 100 m from the farm edge and in an area that had never been farmed). These three classifications represent an ordinal ranking of potential impacts, with the latter acting as a reference. Adjacent sites were chosen to be as close as possible to farm sites without necessarily being in immediate contact with them due to the placement of impediments like netting and poles. The FAR sites were a minimum of 100 m to ensure maximum separation from SF and ADJ sites. Additionally, because of the varying layout of the monolines within farms, farm size was estimated from the location of the mangrove stakes that demarcated their perimeters. Five transects were completed within each site, each measuring 20 m x 5 m to reflect local visibility conditions. Transects were laid parallel to the reef to control for depth which ranged from 0.0m at mean low tide to 2.0m at mean high tide.

**Table 1:** Summary of sampling locations, in increasing order of farm size.

Location	Farm size (m^2^)	Blast	MPA established*	Enforced MPA	Distance to port (km)	Island size (ha)	Pop size (#)
Busili-an	500		2007	✓	4.93	4.28	1654
Pinamgo	500	✓	2002		4.93	4.28	1654
Pandao	600		2002		3.88	0.01	0
Guindacpan	625	✓	1996		9.39	0.13	2204
Tahongtahong	700	✓	N/A		13.4	0.01	200
Saag	750		1997		6.54	0.36	640
Pandanon	900	✓	1996		10.5	0.29	2062
Cataban	1050		1996	✓	6.58	0.7	1251
Bansaan	1050		1994		7.17	1.17	1500
Banbanon	1225		2002		6.62	0.6	0
Jandayan Sur	1300		2002		0.85	4.52	2481
Jandayan Norte	1450		2002		1.88	4.52	2481
Handumon	1500		1995	✓	3.5	4.52	2481
Tambo	2025		N/A		4	1.25	150

*Source http://acccoast.bmb.gov.ph/database/mpa-database

Attributes include the presence/absence of marine protected areas (MPAs), the date of MPA establishment (where applicable), the presence/absence of blast fishing, the size of the seaweed farm and its distance to the nearest port, and the size of, and number of residents on, the associated island (Pop. size).

Locations were classified with respect to the presence of MPAs (MP), the absence of MPAs and blast fishing (“unprotected” or UP), or the presence of blast fishing (BL), with additional information compiled on farm size, distance to market, island area, and population (Table 1). Information on the history of MPAs was obtained from local community members, government representatives, and NGO databases. At these four locations, the no-take MPAs served as the reference (FAR) sites. There were a total of seven locations with neither effective MPAs nor blast fishing (UP). The remaining four locations had extensive levels of blast fishing (both historical and current), based on fisher reports, which were later confirmed by visual evidence (blast craters or directly witnessing the blast fishing itself). The classification of sites as experiencing blast fishing did not include the use of small blasting caps employed by farmers to drive rabbitfish from farms as this practice neither destroys habitat nor kills the fish.

Transect starting point coordinates were assigned using a random number generator and transects were separated by a minimum of 5 m. Transects were only conducted if visibility allowed for clear sight of at least 5 meters forward and 5 meters wide. We used free diving techniques to maximize survey time without requiring SCUBA apparatus, which presented a risk of diver entanglement within seaweed farms. Fish surveys were conducted ten minutes after the line was laid, and passes along transects were timed to maximise consistency. Surveys were generally undertaken in the first pass along transects [37,38], unless large numbers of fish were detected. In these cases, two passes were performed: the first to identify more mobile species, the second focusing on the more sessile/cryptic ones. Individual fish within the belt transect were identified to species level and body lengths were estimated based on training sessions with metal cut outs near sample sites prior to the census [39]. The same investigator conducted all fish transects.

### 2.3 Animal Ethics

All UVC data were collected in accordance with the University of British Columbia (UBC) Policy # 91 (Research and Teaching Involving Animals) and received the approval of the UBC Committee on Animal Care (approval # A10-0158). Permissions for UVC protocols were not required per Philippine Bureau of Fisheries and Aquatic Resources (BFAR) and field studies did not involve endangered or protected species. Studies were conducted in an arc between 9°56’31.77”N, 123° 49’47.09”E and 10°15’37.01”N, 124°39’40.74”E.

## Analysis

Univariate attributes of the fish assemblage included the number of species, total fish abundance, and total fish biomass. While species numbers and total abundance were estimated directly from the UVC, biomass values were derived from species-specific length-weight relationships whereby the weights of individual fish (or a similarly sized congener or confamilial, where unavailable) were calculated from *in situ* estimates of lengths and then summed [40]. Values of species richness, total abundance, total biomass, and individual species’ abundances per transect were then averaged at the site level (SF, ADJ, FAR) for each location to generate mean estimates transect^-1^.

We tested for the effects of farming (SF, ADJ, FAR) and disturbance (MP, UP, BL) on univariate and multivariate attributes of the fish assemblages using permutational techniques [41,42]. Given the sampling occurred during the summer period of two consecutive years at 1 (Handumon) and 13 locations respectively, we reran all analyses without Handumon to test for the potential of this location to influence the results, either because of its innate differences or because of the earlier sampling period. Because the effects of blast fishing and MPAs are likely to occur at the scale of locations (~2.3 km^2^) rather than sites (<2500 m^2^), the analysis was conducted at the former spatial scale. Specifically, Russ (2003) demonstrated spillovers from effective MPAs and Fox (2003) and Fox and Caldwell (2006) have documented the spatially extensive impacts of blast fishing. A nested PERMANOVA was used to test the effects of seaweed farming and disturbance on the univariate metrics of species richness, abundance, and biomass, and on the multivariate relative abundance data. We chose a design where location (LOC), a random factor with 14 levels, and the degree of seaweed farming present (FARM), a fixed factor with three levels, were nested in the amount of human disturbance present (DIS), also a fixed factor with three levels [43]. We tested for the effects of dispersion in the PERMANOVAs using PERMDISP [44,45]and found no significant effects. Where results for PERMANOVA were significant, pairwise tests among levels of FARM were conducted [46]. The univariate analyses were based on a Euclidean distance matrix with no variable transformation [44]. For multivariate data, we used a square root transformation on species abundances to reduce the influence of relatively abundant species and then calculated the Bray Curtis dissimilarity matrix. Additionally, an unconstrained principal coordinate analysis (PCO) was also run using the distances among centroids to visualize both the relative size of the effects and the interactions contained in the multivariate model. Similarity percentage (SIMPER) analyses were conducted to identify the key species distinguishing assemblages as a function of farming and disturbance. Specifically, we identified the top five species that most contributed to dissimilarity between all pairwise combinations of significant factors. All analyses were run in software PRIMER v6.0 [41,44].

## Results

We sampled 210 transects at 42 sites within the 14 locations, capturing data on 80,186 individual fish representing 143 species from 38 families. Fish lengths varied from 1 cm to 40 cm, and small, reef-associated individuals/species generally dominated the assemblage.

### 4.1 Species richness, abundance and biomass

**Fig 2:**
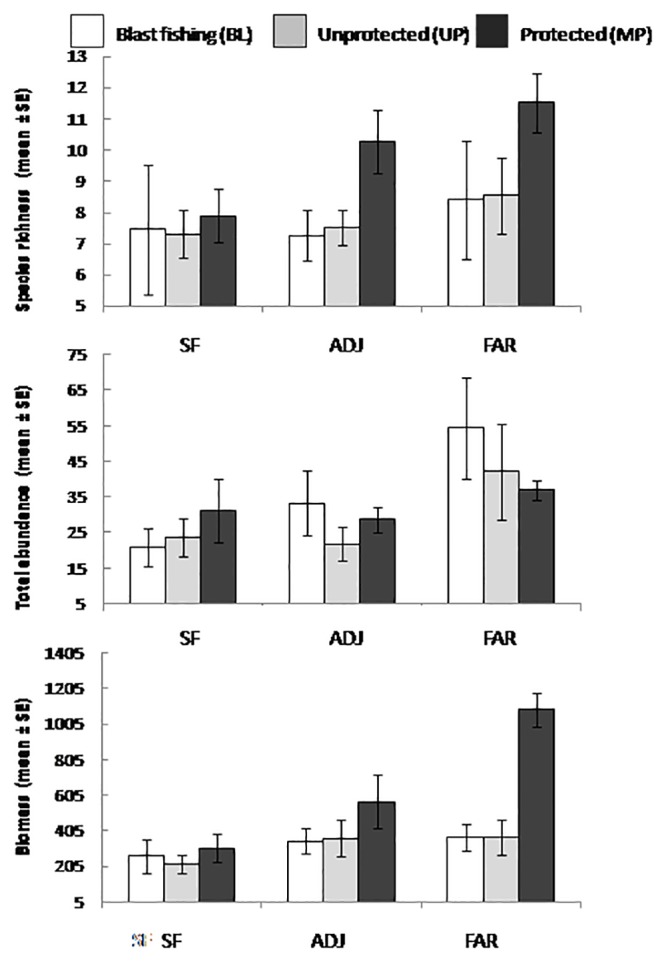
Impacts of human disturbance on the abundance, biomass and diversity of reef-associated fish in the Danajon Bank. SF indicate sites where seaweed farming occurs, ADJ and FAR are adjacent and far sites, respectively. Values represent site-specific averages.

**Fig 3:**
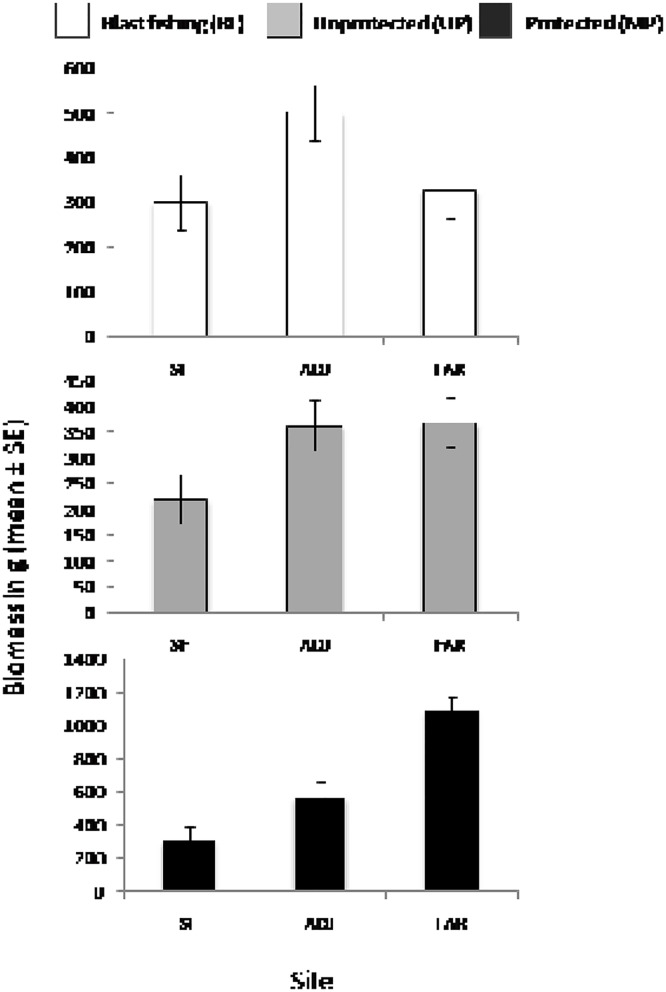
Pairwise tests for Biomass in grams for three levels of disturbance by level of farming present (FARM) where; SF indicate sites where seaweed farming occurs, ADJ and FAR are adjacent and far sites, respectively.

Species richness, averaged by site, varied from 5.1 to 11.3 transect^-1^ across the 14 locations, with a mean of 8.19 (SE = 1.82). Total abundance and biomass ranged from 17.2 to 96.9 transect^-1^ and 93.1 to 916.6 g transect^-1^ respectively, with means of 35.1 transect^-1^ (SE = 20.71) and 441 g site^-1^ (SE = 230). For all three attributes, there was a significant interaction between the effects of farming and location, indicating that the specific nature of how farming affected fish assemblages depended on location (Table 2). On an overall basis as a function of level of disturbance, species richness is greatest in FAR sites but similar in SF and ADJ sites, with the protected locations showing the strongest trends (Fig 2). In the case of abundance, unprotected locations and those subject to blast fishing show the strongest trends, with higher abundances in FAR sites, and no trends in locations with MPAs. Finally, biomass shows similar patterns to species richness with locations with MPAs showing highest biomass in FAR sites (Fig 2). Despite these significant interactions, pairwise tests showed significant results only for biomass within levels of disturbance: there was no affect of FARM on biomass in locations subject to blast fishing and biomass generally increased with distance in UP and MP locations (Fig 3). There was no change to the significance of tests when the Handumon location, sampled in 2010, was excluded from the analysis.

**Table 2:** Univariate permutational analysis of variance (PERMANOVA) of the effects of seaweed farming (FARM) and human disturbance (DIS) on fish species richness, abundance and biomass.

	Source	P(perm)	df	SS	MS	Pseudo-F
Species richness	DIS	NS	2	218.61	109.30	3.11
	FARM(DIS)	NS	6	137.94	22.99	0.90
	LOC(DIS)	1.00E-05	11	384.64	34.97	6.99
	FARM(DIS)xLOC(DIS)	1.00E-05	21	540.20	25.72	5.14
	Residuals		169	845.8	5.0047	
Abundance	DIS	NS	2	714.49	357.25	0.16
	FARM(DIS)	NS	6	15833.00	2638.80	1.34
	LOC(DIS)	0.002	11	24953.00	2268.40	3.27
	FARM(DIS)xLOC(DIS)	0.001	21	41476.00	1975.00	2.85
	Residuals		169	1.17E+05	693.41	
Biomass	DIS	0.02	2	3.73E+06	1.86E+06	6.07E+00
	FARM(DIS)	0.0034	6	5.41E+06	9.01E+05	4.69E+00
	LOC(DIS)	0.005	11	3.36E+06	3.05E+05	2.54E+00
	FARM(DIS)xLOC(DIS)	0.05	21	4.05E+06	1.93E+05	1.61E+00
	Residuals		169	2.03E+07	1.20E+05	

Sampling locations were either subject to blast fishing (BL), unprotected(UP) or protected (MPA). Within these locations, three types of sites were examined: seaweed farm sites (SF), adjacent sites (ADJ) and far sites (FAR). Location (LOC) is a random factor nested in both FARM and Disturbance, which are fixed factors, reporting degrees of freedom (df), sums of squares (SS), mean squares (MS), F values and p. All Pairwise tests significant at p<0.001.

### 4.2 Species assemblage composition

**Fig 4:**
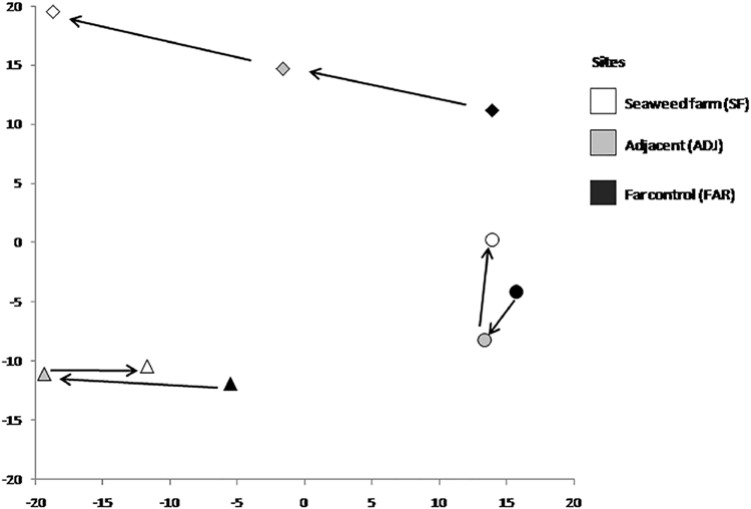
Principal coordinates analysis (PCO) showing the centroids for Location (MPA = diamond; and UP = circle; BL = triangle; and FARM levels (SF = black; ADJ = grey; and FAR = white).

Species assemblage composition varied significantly the level of disturbance (MP, UP, BL) among islands (p = .0001) and there was a significant interaction between the level of farming which was present (SF, ADJ, FAR) and the location as a function of the level of disturbance (p = 0.0001) (Table 3). The greatest differences in assemblage structure within locations were for those locations with MPAs, where a directional gradient from seaweed farms to sites distant from the farms could be observed (Fig 4). Locations without MPAs, whether subject to blast fishing or not, had clear differences in their assemblages between the effects of seaweed farms, but lacked clear directionality (Fig 4).

**Table 3:** Multivariate permutational analysis of variance (PERMANOVA) of the effects of seaweed farming (FARM) and human disturbance (DIS) on fish species richness, abundance and biomass.

	Source	P(perm)	df	SS	MS	Pseudo-F
Assemblage	DIS	0.16	6	34079	5679.8	1.182
	FARM(DIS)	0.25	2	50086	25043	1.1734
	IS (DIS)	0.0001	11	2.33E+05	21215	11.052
	FARM(DIS)xIS (DIS)	0.0001	21	1.01E+05	4824.6	2.5134
	Residuals		173	3.24E+05	1919.6	

Sampling locations were either subject to blast fishing (BL), unprotected (UP) or protected (MPA). Within these locations, three types of sites were examined: seaweed farm sites (SF), adjacent sites (ADJ) and far sites (FAR). Location (LOC) is a random factor nested in both FARM and Disturbance which are fixed factors, reporting degrees of freedom (df), sums of squares (SS), mean squares (MS), F values and p. All Pairwise tests significant at p<0.001.

**Fig 5:**
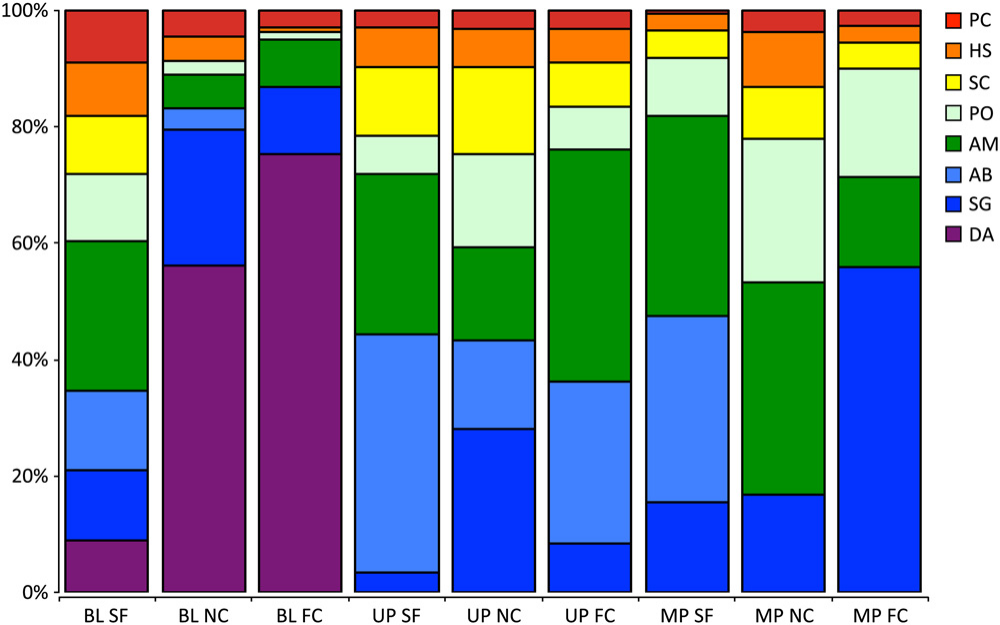
Simper of total abundance for top eight species present at each of 14 locations: *Amblypomacentrus breviceps* (AB), *Apogon margaritophorus* (AM), *Dascyllus aruanus* (DA), *Halichoeres scapularis* (HS), *Pomacentrus chrysurus* (PC), *Pomacentrus opisthostigma* (PO), *Siganus canaliculatus* (SC), and *Scarus ghobban* (SG), where locations were ranked along a gradient of disturbance from: blast fishing with no protected area present (BL), no blast fishing but no protection (UP), and no blast fishing with a protected area present (MP).

We identified eight species that corresponded to the effects of farming and disturbance. These included small species such as the damselfishes *Amblypomacentrus breviceps*, *Dascyllus aruanus*, *Pomacentrus chrysurus* and *Pomacentrus opisthostigma*, the cardinal fish *Apogon magaritiphorus*, and a small wrasse *Halichores scapularis*. Medium grazers such as the parrotfish *Scarus ghobban* and the rabbitfish *Siganus canaliculatus*, both of which are also important target species, also corresponded to the effects of farming and disturbance. Specifically, distinct differences were evident in the abundance of these indicator species, separating the locations with blast fishing from those without. In locations where blast fishing was present, the assemblage was dominated by *D*. *aruanus*, which feeds on plants and invertebrates and tends to inhabit isolated coral heads in small groups [40]; its numbers systematically declined with proximity to seaweed farms. Additionally, *A*. *magaritiphorus*, a small omnivorous cardinal fish [40] was also present in blast fishing locations but was also more common in seaweed farms. *Amblypomacentrus breviceps*, known to frequent rubble in sand or silty areas [40], was also common in seaweed farms regardless of the presence or absence of blasting (Fig 5). Of the two medium sized species, *S*. *canaliculatus*, an obligate herbivore, was present only in farmed sites in locations subject to blast fishing, while *S*. *ghobban*, a grazer known to feed on both detritus and plants [40], was most common in locations with MPAs and more common in the near controls of locations subject to blast fishing or unprotected (Fig 5).

## Discussion

Our objective was to understand how seaweed farms influence shallow coral fish assemblages, given the increasing fragmentation of seascapes driven by this rapidly growing sector. The expansion of seaweed farms on the Danajon Bank is also occurring in the context of MPA establishment and ongoing destructive practices such as blast fishing, which result in a complex and constantly evolving matrix of human use. We hypothesized that seaweed farms may generate ecological benefits by creating habitat and providing a food source for other fishes [8] in otherwise generally degraded seascapes [47]. Specifically, because seaweed farms potentially added both habitat structure and a food source, we predicted that the fish assemblages would exhibit greater diversity, abundance, and biomass in closer proximity to seaweed farms. Our results generally suggested the opposite, with species richness and total biomass tending to decline with proximity to seaweed farms in the absence of blast fishing and total abundance showing the same effect where blast fishing was present. These results were consistent with previous studies on seaweed farms established on seagrass communities [10,12] and with patterns observed in other regions where habitat homogenisation occurred [5,48–50]. Specifically, seaweed farms located in seagrass habitats negatively alter macrofaunal community composition as well as large invertebrate epifauna and fish communities [10,12]. In homogenized habitats, the further loss of ecological function can actually be proportionally greater than expected from simply an overall decline in species richness, possibly due to the fact that those species that are lost tend to be non-randomly distributed among functional and ecological categories, but tend to include larger specialist species and therefore have a disproportionately large effect on the physical and biological environment [49–51]. The fact that FAR sites typically had greater biomass than seaweed farms and their adjacent areas may indicate that the latter lack suitable habitat/cover for these animals and/or that the level of human activity within and adjacent to the farms is sufficiently disruptive to drive fish away. Reported impacts of seaweed farms on coral habitat include trampling, shading and siltation [8]. Such disturbances could potentially have a range of effects including the fragmentation, degradation or loss of preferred habitat [52]. As many reef associated fishes display specific habitat requirements, such as food sources, recruitment habitat [53], or topographical complexity (e.g. holes, crevices, occupying caves etc..) [54], seaweed farms have the potential to negatively affect both the biological and physical structure of shallow coral reefs, which in turn may result in a loss of diversity, and decreases in abundance and biomass [49].

The effect of farming was greatest in those locations with effective MPAs. These locations tended to have higher biomass within the MPA (FAR sites) relative to the associated seaweed farms and adjacent sites, with abundance showing less clear patterns. Such patterns are consistent with those typically reported for MPAs, where species richness, and biomass are highest inside, but then decline with increasing distance from the MPA [55–57]. Moreover, the scale of the relative differences between FAR sites and seaweed farms was also consistent with previous MPA studies: species richness was approximately 30% greater in the MPAs relative to typical reports of 20–30% increases and biomass was 300% higher relative to unprotected areas [58]. It is therefore likely that the observed increases in total biomass with distance from the seaweed farms were due to the beneficial effects of the MPAs in the FAR sites rather than just a negative effect of the seaweed farms at these locations. Unprotected locations and those with blast fishing had lower diversity and biomass than did locations with MPAs, and indeed, were relatively indistinguishable which is consistent with anticipated effects of increased habitat homogenisation associated with increased levels of disturbance [49–51,59]. The exception to this pattern appeared in FAR and ADJ sites where total abundance was higher in both unprotected and blasting locations. This was due to their respective assemblages being dominated by numerous, small-bodied fish like *A*. *magaritaphorous*, which was commonly found among patches of rubble and debris (Hehre pers. obs.).

Abundance showed similar patterns to species richness and biomass at locations without MPAs. In these locations, abundance was on average 1.8 and 2.6 times greater in FAR sites than in seaweed farms in unprotected and blast fishing locations respectively. This was an unexpected result because the habitats in locations subject to blast fishing or unprotected were generally severely degraded (Hehre, pers. obs.). The high abundance at FAR sites at blast locations was due to high numbers of the small damselfish *D*. *aruanus*, which has an affinity for live branching coral and a dislike of disturbed reef habitat [60,61]. However, it was notable that the *D*. *aruanus* was exclusively observed in high numbers on the last remaining pieces of branching coral in these locations (Hehre pers. obs.). This may be due in part to several coinciding factors including that once seaweed farms are in place, blast fishers avoid the farms and their general vicinity due to social constraints [8]. This was evident in communities on the Danajon Bank where blasting was prevalent, but considerable care was taken to avoid damaging neighbouring seaweed farms (Hehre pers. obs.). In locations with blast fishing, seaweed farms also tended to be heavily cleared and the coral rubble piled outside the perimeter of the seaweed farm (Hehre pers. obs.). In these locations, the displaced coral rubble was frequented by large numbers of small fish such as *A*. *magaritaphorous*, which find themselves sandwiched between two structurally degraded environments, one the result of blast fishing [59] and the other the result of intentional clearing.

Univariate and multivariate analysis were in good agreement, and showed that species composition varied most with level of disturbance, as evidenced by the strong separation of locations between those with MPAs, those that are unprotected and those subject to blast fishing. In locations with MPAs, the shift in fish assemblage from small species such as *A*. *margaritophorus* in the seaweed farms to larger target species such as the parrotfish *S*. *ghobban* in the MPAs is consistent with effective protection of target species and increase in mean size [57,58]. The lack of directional differences in the assemblages of unprotected and blast locations may reflect the relatively depauperate composition of these locations, such that differences between sites as a function of distance from seaweed farms were difficult to discern [62]. Fish assemblages within the Danajon Bank are under continual disturbance from a variety of anthropogenic sources including destructive fishing practices, clearing, trampling, and exposure to pollution [8,55]. These locations may simply have reached a resultant level of habitat degradation where the only constituent members of the fish community to fill this niche are the few species that can tolerate high levels of anthropogenic disturbance [49,50].

We identified a set of eight indicator species that distinguished sites on the basis of seaweed farming and the presence/absence of blast fishing and MPAs, out of the 143 species recorded. That only eight species accounted for the vast majority of differences between locations potentially reflects the already relatively species-reduced nature of the region [47]. Locations subject to blast fishing were characterised by very small species such as *D*. *aruanus*, *A*. *magaritaphorous*, *P*. *bifasciatus* and *P*. *opisthostigma*. This pattern is consistent with other studies that document the role of blast fishing in decreasing structural complexity of coral reefs, thereby favouring dominance by small, disturbance-tolerant species [59,63,64]. Two of our indicator species, *S*. *ghobban* and *S*. *canaliculatus*, are relatively large bodied herbivores that are highly sought as food fish on the Danajon Bank [65], and were more common at MPA sites. Of particular interest was *S*. *canaliculatus*, as seaweed farmers report that this rabbitfish forages heavily on farmed seaweeds, likely due to their reported obligate herbivory [66]. Accordingly, we had expected to see increased numbers associated with seaweed farms, however this was not reflected in the data. The lack of rabbitfish in the seaweed farms may reflect both the use of small blasting caps to drive away this perceived crop pest and the quite significant pressure ensuing from targeted spearfishing (Hehre, pers. obs.). It may be that fishing pressure is sufficient to maintain low numbers of rabbitfish even within seaweed farms despite their potential as a supplementary food source.

These results have significant implications for seascape management. First, we found no evidence to suggest that seaweed farms have the potential to generate benefits with respect to fish assemblages. Moreover, species richness and biomass decreased with proximity to seaweed farms even in locations subject to blast fishing or that are otherwise unprotected. This suggests that there may be a net negative effect of seaweed farms on fish assemblages despite the already generally degraded nature of these shallow coral reef ecosystems. Second, our study supported the importance of MPAs for fish in shallow coral habitats since the presence of MPAs was the most influential determinant of species richness and biomass. Both points are particularly important because seaweed farms currently bound no-take MPAs and there have been proposals to include seaweed farms within no-take MPAs, rendering them multiple-use MPAs. Specifically, some of these proposals involve using seaweed farms as physical buffers against destructive fishing practices as well as integrating seaweed farming within the boundaries of MPAs in order to potentially gain political consensus for the expansion of protected areas. Seaweed farming has typically been perceived as “ecologically friendly”, with minimal negative effects on fish or benthos in already degraded ecosystems with the added benefit that the presence of farm workers can act as a means of MPA enforcement. We argue that seaweed farms can have a negative impact on fish assemblages and that fish within MPAs, as currently enforced, are more diverse, abundant and larger than those observed in farms. To this end, the placement of seaweed farms should be carefully and cautiously considered, with particular attention being drawn to farm sites adjacent to, or located within, MPAs.

The regional scale of our study allowed us to test the effects of seaweed farms on fish assemblages against existing practices that include the over-exploitation of fisheries resources, the presence of destructive fishing [17], and the positive introduction of MPAs. Seaweed farming has become an important source of income to families dependent on increasingly sparse catch returns [67]. However, in spite of the initial promotion of seaweed farming as an ecologically friendly alternative livelihood, there remains little evidence that farming mitigates fishing pressure [67], or provides benefits for constituent shallow coral fish assemblages. At present few environmental conditions are considered in either the number of permits issued for seaweed farms, or in their location on shallow coral ecosystems. In this context, our results have implications for managers who need to both address the need for alternative livelihoods given unsustainable fishing practices and find ways of maximizing the positive benefits of MPAs whilst minimizing the negative effects of seaweed farms.
